# Occupational characteristics and epigenetic aging among older adults in the United States

**DOI:** 10.1080/15592294.2023.2218763

**Published:** 2023-06-10

**Authors:** Theresa Andrasfay, Eileen Crimmins

**Affiliations:** Leonard Davis School of Gerontology, University of Southern California, Los Angeles, CA, USA

**Keywords:** DNA methylation age, epigenetic clock, epigenetic age acceleration, pace of ageing, working conditions, occupation

## Abstract

Occupational characteristics have been studied as risk factors for several age-related diseases and are thought to impact the ageing process, although there has been limited empirical work demonstrating an association between adverse occupational characteristics and accelerated ageing and this prior work has yielded mixed results. We used the 2010 and 2016 waves of the Health and Retirement Study (*n* = 1,251) to examine the association between occupation categories and self-reported working conditions of American adults at midlife and their subsequent epigenetic ageing as measured through five epigenetic clocks: PCHorvath, PCHannum, PCPhenoAge, PCGrimAge, and DunedinPACE. We found that individuals working in sales/clerical, service, and manual work show evidence of epigenetic age acceleration compared to those working in managerial/professional jobs and that the associations were stronger with second- and third-generation clocks. Individuals reporting high stress and high physical effort at work showed evidence of epigenetic age acceleration only on PCGrimAge and DunedinPACE. Most of these associations were attenuated after adjustment for race/ethnicity, educational attainment, and lifestyle-related risk factors. Sales/clerical work remained significantly associated with PCHorvath and PCHannum, while service work remained significantly associated with PCGrimAge. The results suggest that manual work and occupational physical activity may appear to be risk factors for epigenetic age acceleration through their associations with socioeconomic status, while stress at work may be a risk factor for epigenetic age acceleration through its associations with health behaviours outside of work. Additional work is needed to understand when in the life course and the specific mechanisms through which these associations occur.

## Introduction

A common adage is that working in a particularly stressful or demanding job ages one prematurely. This is supported by anecdotes such as the early greying of presidents’ hair, but beyond these anecdotes about aesthetic aspects of ageing, it is unclear whether demanding work is associated with accelerated ageing at the molecular level. Understanding whether individuals in certain jobs age faster than others is important to understand the process through which work affects health at older ages.

One way to assess the pace of ageing is through epigenetic age acceleration. Gene expression can be influenced by the addition of methyl groups to CpG sites, locations of the DNA where cytosine is followed by guanine [1]. Because certain CpG sites have predictably increased or decreased methylation with age, the amount and location of DNA methylation (DNAm) can be used to construct summary measures of ageing, often called epigenetic clocks because they reveal how much time has elapsed according to one’s epigenome [2,3]. Epigenetic age acceleration (EAA) refers to the difference between epigenetic age and chronological age, and positive EAA is associated with markers of social disadvantage and is predictive of mortality and the onset of several age-related diseases [2,4–10].

Several epigenetic clocks have been developed. First generation clocks, including the Horvath and Hannum clocks, were early measures developed to predict chronological age from DNAm patterns [11,12]. While first-generation clocks were strongly associated with chronological age, they were not consistently associated with other age-related outcomes, so researchers developed second-generation clocks to predict morbidity and mortality [2,3]. For example, PhenoAge was trained on a phenotypic age estimated from chronological age and nine age-related physiologic biomarkers [13], while GrimAge was developed by first identifying DNAm markers of plasma proteins and lifetime smoking and then utilizing these DNAm markers in combination with chronological age and sex to estimate remaining lifespan [14]. Last, DunedinPoAM and the more-recent DunedinPACE measures can be described as third-generation measures that capture the rate of epigenetic ageing, as opposed to current epigenetic age, because they are trained on trajectories of age-related biomarkers [15].

Occupational characteristics have been studied in relation to numerous health outcomes at older ages, with research indicating that individuals with less favourable working conditions tend to have increased risk of age-related health outcomes. Compared to those in professional or managerial occupations, individuals in both manual and service work have worse self-assessed overall health [16,17], and individuals in manual work have increased risk of hypertension [18]. Psychosocial stressors at work have been associated with lower cognitive functioning and diabetes [19,20], while physical demands at work have been associated with reduced physical and cognitive functioning [21,22], and long working hours have been associated with increased risk of coronary heart disease [23]. In addition to these associations with health conditions at older ages, service work, psychosocial job stress, physical demands, and long working hours have been associated with accelerated biological ageing as measured through physiologic biomarkers, suggesting these working conditions may influence an underlying ageing process [24].

Until recently there has been limited research examining the association between occupational characteristics as risk factors for EAA. Prior studies have examined occupational position or prestige as it relates to epigenetic age, with several finding that individuals in lower ranked occupations have positive EAA, especially when measured with second-generation clocks [6,10,25,26]. With the exception of Freni-Sterrantino et al. [25,26], these studies have typically treated occupation as a component of or proxy for socioeconomic status. While occupation is an important component of socioeconomic status, it also includes a set of conditions and exposures that can influence one’s health, and from these previous studies it is unclear whether it is the types and characteristics of jobs or the job’s association with other aspects of socioeconomic status that should be considered risk factors for accelerated ageing. Research assessing the associations between specific working conditions and epigenetic ageing is also limited, though studies in Finland and the United Kingdom have found long working hours, psychosocial job strain, and physical job demands to be associated with accelerated epigenetic ageing [25–27].

The objective of this study is to examine how both occupational categorizations and self-reported working conditions are associated with EAA and how these associations are modified by adjustment for social background characteristics and lifestyle-related risk factors for EAA. Occupational categorizations may better proxy for one’s socioeconomic background and status while working conditions provide information on the specific tasks or stressors faced at work, and the consideration of both is important to understand which aspects of work should be considered risk factors for accelerated ageing. In this study, we use data from the Health and Retirement Study (HRS) to examine these associations in a population-based sample of older American adults employed in a wide range of occupations and industries.

## Data

**Figure 1.**
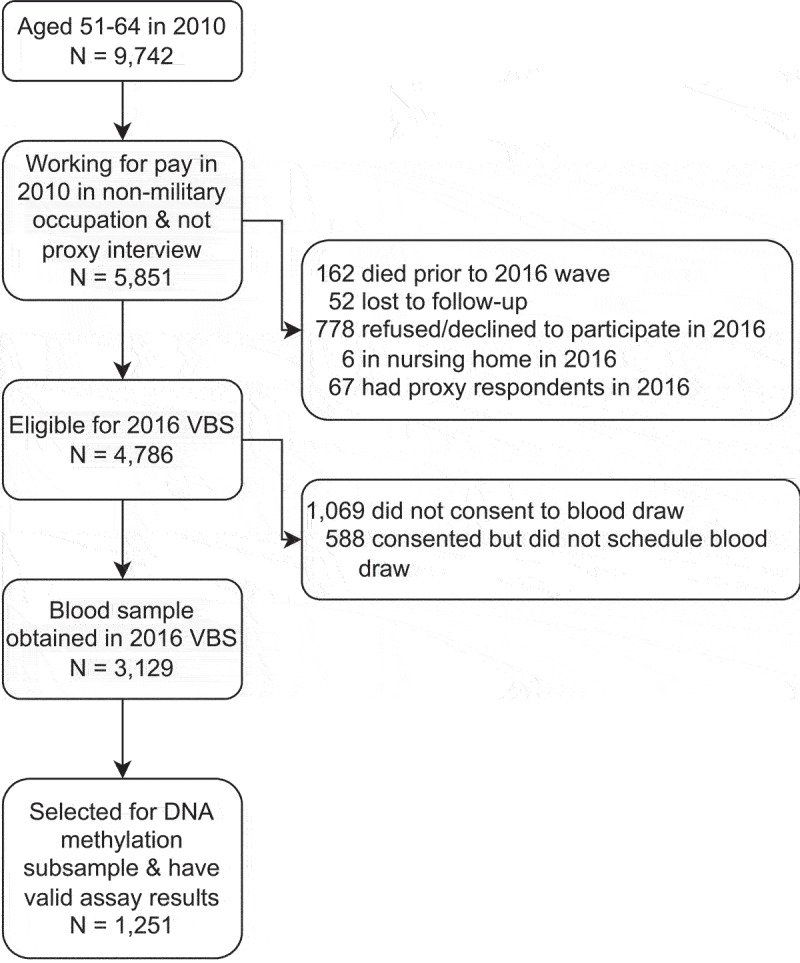
Sample selection diagram. Data are from the health and retirement study. VBS = Venous blood study.

Data are taken from the Health and Retirement Study (HRS), a nationally representative panel study of older adults in the United States. The HRS (Health and Retirement Study) is sponsored by the National Institute on Aging (grant number NIA U01AG009740) and is conducted by the University of Michigan. In 2016, the HRS added the Venous Blood Study (VBS), which included DNAm assays for a representative subsample of the VBS participants [28]. VBS participants were aged 56 and over when DNAm was measured in 2016. To obtain information on occupational characteristics of these participants at younger ages, we pull information on occupational characteristics from the 2010 wave, when these participants were aged 51 and over. We further restrict our sample to individuals under age 65 in 2010 to limit the effect of selection out of the labour force when individuals reach typical retirement and Medicare eligibility ages. We also limit our analyses to individuals working in non-military occupations, as military occupations are not easily categorized into the occupation categories used in our study. The sample selection process is summarized in Figure 1 and results in an overall analytic sample of 1,251 individuals.

These data are available to registered users who meet security requirements and agree to data use conditions specified by the HRS. The secondary analysis of these data for this study was approved by the Institutional Review Board at the University of Southern California.

### Measures

#### Epigenetic age

As part of the HRS VBS in 2016, phlebotomists conducted in-home visits to VBS participants and collected six blood samples, including one in a 10 mL EDTA whole blood tube, from which DNA was extracted. DNAm was assayed using the Infinium Methylation Epic BeadChip (v1.0), with samples randomized across plates by demographic variables. All sample processing and assays were conducted by the Advanced Research and Diagnostic Laboratory at the University of Minnesota. Further details on the data collection and processing are available from the HRS [28]. From the DNAm data, epigenetic clocks were estimated and made available to researchers [28].

Of the 13 epigenetic age measures available for VBS participants, we chose five that have been analysed in the previous literature examining occupational characteristics or socioeconomic position as predictors of EAA and that have been associated with the types of ageing-related outcomes we would expect to be related to occupational characteristics. These include Horvath DNAmAge, Hannum DNAmAge, PhenoAge, GrimAge, and Dunedin PACE. With the exception of the Horvath clock, these clocks are not adjusted for blood cell composition, so they implicitly take into account age-related changes in immune functioning and can be considered to measure ‘extrinsic ageing’ [2]. This analysis uses the ‘PC’ versions of epigenetic clocks estimated through principal components analysis, which improves the reliability of these estimates [29]. These PC clocks were estimated from the DNAm data by the Levine Lab [29].^1^

#### Occupational characteristics

HRS respondents who report currently working for pay are asked a series of questions about their main job in the HRS core interviews. We use occupation codes, derived from respondents’ reports about their job titles or job descriptions, to classify respondents’ occupations into four broad categories: managerial/professional, sales/clerical, service, and manual. We also utilize respondents’ answers to questions about the amount of stress involved at work, physical effort required, and their typical hours worked per week at their main job. We dichotomize these self-reported working conditions into high stress for respondents reporting they strongly agree (as opposed to agree, disagree, or strongly disagree) that their job involves lots of stress, high physical effort for respondents reporting that their job requires lots of physical effort all or almost all the time (as opposed to most of the time, some of the time, or none or almost none of the time), and long working hours for respondents working at least 55 hours per week, a threshold that has been shown in prior studies to be associated with adverse health outcomes [30].

#### Covariates

Demographic characteristics include sex and chronological age in 2016, the same year in which epigenetic age is measured. We also consider two social factors that are associated with selection into occupations and have been associated with epigenetic ageing in prior work: educational attainment and race/ethnicity. We categorize educational attainment as college degree, some college, high school or equivalent, and less than high school, and race/ethnicity as non-Hispanic white, non-Hispanic Black, Hispanic, and non-Hispanic other. Last, we include four lifestyle-related risk factors for epigenetic ageing that are measured in 2016. Smoking status is categorized as never smoker, former smoker, or current smoker. Alcohol consumption is categorized as non-drinker, moderate drinker, which is defined as drinking at most one drink per occasion for women and at most two drinks per occasion for men, and more than moderate drinker, which is defined as drinking in excess of the cut-offs for moderate drinking. Physical inactivity is defined as hardly ever or never engaging in either moderate or vigorous physical activity. Body mass index (BMI) is categorized as healthy weight or underweight (BMI <25), overweight (25 ≤ BMI <30), obese class I (30 ≤ BMI <35), and obese class II or II (BMI ≥ 35).

#### Analysis

We conduct three sets of analyses to estimate the associations of the five epigenetic age measures with a) occupational categorizations, b) self-reported working conditions, and c) both occupational categorizations and self-reported working conditions. For each set of analyses, we estimate three nested linear regression models (ordinary least squares) that are theoretically informed by our objectives for this analysis. Our first objective is to establish differences in epigenetic age measures by occupational characteristics after standard demographic adjustments for age and sex and for this reason, Model 1 includes the occupational characteristics, chronological age in 2016 and sex. The second objective is to assess whether differences in epigenetic age by occupational characteristics persist after adjustment for social background characteristics that are related to selection into occupations and thus may confound the observed associations between occupational characteristics and epigenetic age. Model 2 additionally includes race/ethnicity and educational attainment, which have been associated with EAA [4,8,9,31]. If associations remain significant after adjustment for these factors, occupational characteristics may be independently associated with EAA. Our third objective is to examine potential mechanisms through which occupational differences in epigenetic age measures may occur by including more-proximate behavioural predictors of EAA, including smoking status, alcohol consumption, physical inactivity, and overweight/obesity [4,8,9,31]. Model 3 thus includes the following lifestyle-related risk factors assessed in 2016: smoking status, alcohol consumption physical inactivity, and BMI category. If occupational differences in epigenetic age arise because occupational characteristics influence these lifestyle-related risk factors, we would expect the associations to be attenuated or reduced once we include these more-proximate risk factors in our models.

Because these models include chronological age, the coefficients can be interpreted as the degree of epigenetic age acceleration (EAA) associated with the occupational characteristic. However, when the outcome is DunedinPACE, the coefficients should be interpreted as the change in the pace of ageing per year of chronological age. Because approximately 6% of our analytic sample was missing at least one variable included in the analyses, we used multiple imputation by chained equations to probabilistically impute 10 plausible values of missing items [32]. In addition to the variables contained in the analysis, we included information on longest-reported occupation category, individual earnings, household income, and household wealth in the imputations. The results presented below are pooled estimates across these 10 complete datasets. All analyses include the respondent weights created by the HRS for the DNA methylation subsample.

## Results

Summary statistics of the analytic sample are displayed in Table 1. The average chronological age of sample participants in 2016 is 62.4, and the average ages on the PCHorvath (59.7), PCHannum (61.2), and PCPhenoAge (59.5) clocks are all similar. However, the average age of the PCGrimAge clock (72.1) is nearly a decade higher than the average chronological age. As expected, the pace of epigenetic ageing, as measured by DunedinPACE, is approximately one year for each chronological year. The most prevalent occupation category held by sample members is managerial/professional (44%), followed by sales/clerical (25%), manual (17%), and service (14%). High stress and high physical effort are reported by approximately one-fifth of the sample, while 10% report working at least 55 hours per week.

**Table 1.** Summary statistics of analytic sample: percent or mean (SD).

	Mean (SD) or %	Percent Missing
Number of respondents	1,251	
Chronological age in 2016	62.4 (3.8)	0
Epigenetic clocks in 2016		0
Horvath	59.7 (5.8)	
Hannum	61.2 (6.3)	
PhenoAge	59.5 (6.8)	
GrimAge	72.1 (4.8)	
Dunedin PACE	0.99 (0.14)	
Current occupational category in 2010		<1
Professional/managerial	43.8	
Sales/clerical	24.8	
Service	14.1	
Manual	17.3	
Self-reported occupational characteristics in 2010		
High stress	21.7	<1
High physical effort	17.1	<1
Long hours (55+ hours per week)	10.3	1.3
Covariates		
Sex		0
Male	49.0	
Female	51.0	
Race/ethnicity		<1
Non-Hispanic White	79.3	
Non-Hispanic Black	9.1	
Hispanic	8.1	
Non-Hispanic Other	3.5	
Educational attainment		<1
Less than high school	36.8	
High school or equivalent	10.7	
Some college	46.7	
College or more	5.8	
Smoking status in 2016		<1
Never smoker	45.2	
Former smoker	43.3	
Current smoker	11.5	
Alcohol consumption in 2016		<1
Non-drinker	29.0	
Moderate drinker	49.3	
More than moderate drinker	21.7	
Physical activity in 2016		<1
Active	91.6	
Inactive	8.4	
Body Mass Index in 2016		<1
Underweight/Healthy weight (<25)	23.6	
Overweight (25 to <30)	39.1	
Obese class I (30 to <35)	24.2	
Obese class II/III (≥35)	13.1	

Note: Data are from the Health and Retirement Study. The sample is restricted to participants aged 51–64 in 2010 who reported working for pay in a non-military occupation in the 2010 HRS core wave and who subsequently participated in the 2016 Venous Blood Study (VBS) and were part of the DNA methylation assay subsample. Summary statistics are weighted with VBS DNA methylation sample weights.

### Occupational categorizations

**Figure 2.**
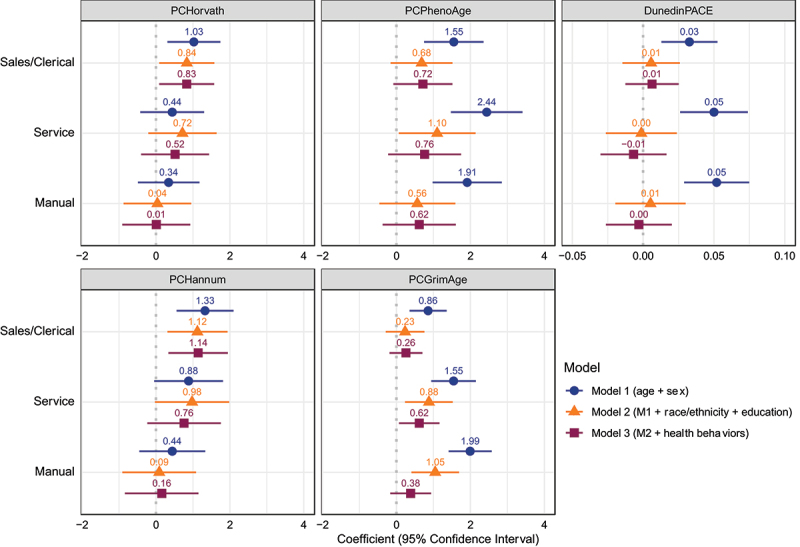
Associations between current occupation category in 2010 and epigenetic age measures in 2016. The displayed effect sizes are the coefficients from linear regressions along with the 95% confidence intervals around these coefficients. The reference occupational category is managerial/professional and the coefficients for the other categories indicate the estimated difference in the epigenetic age measure between that category and managerial/professional after accounting for the other included covariates. Model 1 adjusts for age and sex. Model 2 additionally includes race/ethnicity and educational attainment. Model 3 additionally includes smoking status, alcohol consumption, physical inactivity, and Body Mass Index (BMI) category.

Results from regressions predicting the epigenetic age measures from occupational categories are displayed in Figure 2; additional output from the regressions, including p-values and goodness-of-fit statistics are displayed in tables A1-A3 in Appendix A. The displayed effect sizes are the coefficients from linear regressions along with the 95% confidence intervals around these coefficients. For reference, we include the dashed vertical line at 0 to facilitate distinguishing between statistically significant associations that do not cross this line and nonsignificant associations that cross this line. The reference occupational category is managerial/professional, so the coefficients for the other categories indicate the estimated difference in the epigenetic age measure between that category and managerial/professional after accounting for the other included covariates. For example, in the minimally adjusted model 1, sales/clerical work is significantly associated with 1.03 years older epigenetic age on the PCHorvath clock relative to individuals of the same age and sex who have managerial/professional occupations, but the other occupational categories are not significantly associated with epigenetic age on the PCHorvath clock.

In both the PCHorvath and PCHannum clocks, sales/clerical work is significantly associated with accelerated epigenetic ageing, and this association remains significant after adjustment for both sociodemographic and lifestyle-related risk factors. In the minimally adjusted model predicting PCPhenoAge, sales/clerical work is associated with 1.55 years, service work with 2.44 years, and manual work with 1.91 years of EAA compared with managerial/professional work. Service work remains associated with 1.1 years of EAA in the model controlling for race/ethnicity and education, but none of these categories remain significant after controlling for lifestyle-related risk factors. With PCGrimAge, the minimally adjusted model shows a clear gradient wherein sales/clerical work is associated with 0.86 years, service work with 1.55 years, and manual work with nearly 2 years of EAA compared to managerial/professional work. Both service and manual work remain associated with 0.88 and 1.05 years of EAA in model 2, but only service work remains significantly associated with 0.62-years of EAA in the fully adjusted model. Last, sales/clerical, service, and manual work categories are all associated with a significantly faster pace of ageing on the DunedinPACE measure in the minimally adjusted model, but these associations are no longer significant after adjusting for sociodemographic characteristics.

### Self-reported working conditions

**Figure 3.**
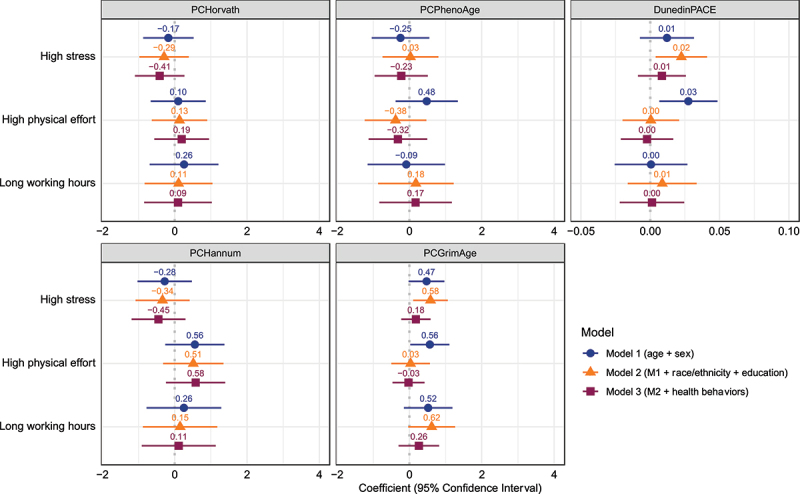
Associations between self-reported working conditions in 2010 and epigenetic age measures in 2016. The displayed effect sizes are the coefficients from linear regressions along with the 95% confidence intervals around these coefficients. These self-reported working conditions are all binary measures and the coefficients refer to the estimated difference in a measure between individuals with and without this occupational characteristic after accounting for the other included covariates. Model 1 adjusts for age and sex. Model 2 additionally includes race/ethnicity and educational attainment. Model 3 additionally includes smoking status, alcohol consumption, physical inactivity, and Body Mass Index (BMI) category.

The next set of models, displayed in Figure 3, examines the self-reported working conditions as predictors of epigenetic ageing. These self-reported working conditions are all binary measures, so the coefficients refer to the estimated difference in a measure between individuals with and without this occupational characteristic after accounting for the other included covariates. These conditions – high stress, high physical effort, and long working hours – are not significantly associated with epigenetic ageing on the PCHorvath, PCHannum, or PCPhenoAge clocks. High physical effort is associated with 0.56 years of accelerated epigenetic ageing on the PCGrimAge clock in the minimally adjusted model, and high stress is significantly associated with 0.58 years of EAA in model 2. However, none of these conditions are significant predictors of PCGrimAge in the fully adjusted model. Last, high physical effort is associated with a 0.03-year per year faster pace of ageing in the minimally-adjusted model and high stress with a 0.02-year per year faster pace of ageing in model 2, but none of the characteristics remain significant in the fully-adjusted model.

### Joint modelling of occupational categorizations and self-reported working conditions

**Figure 4.**
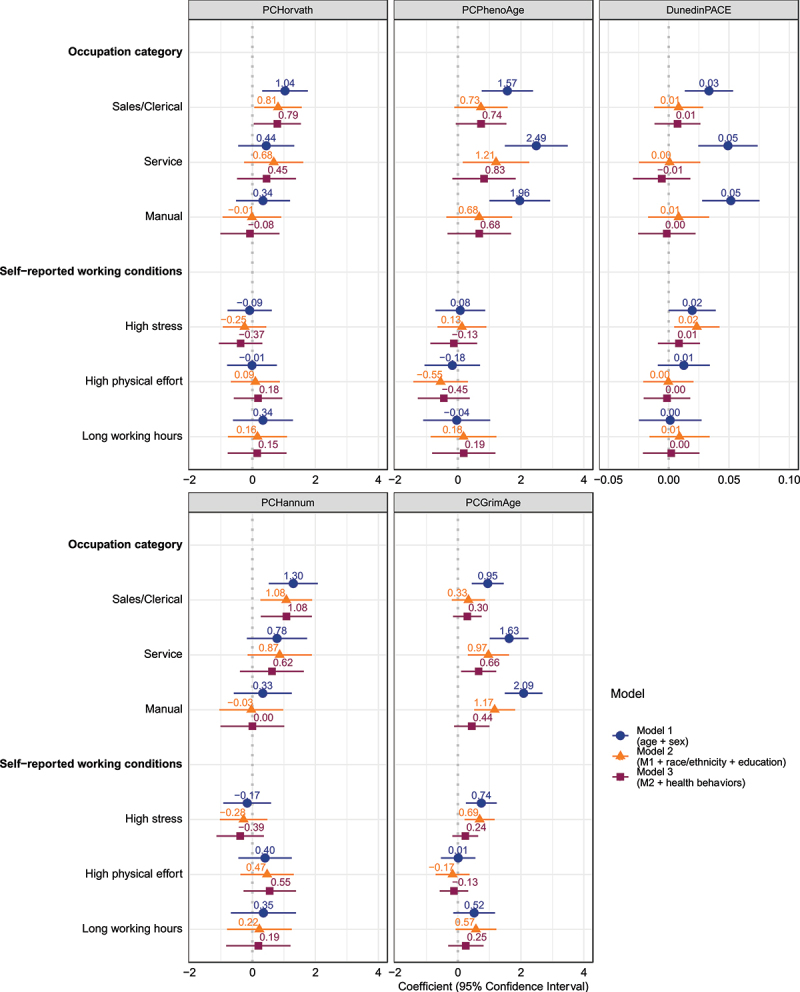
Joint association between current occupation category and self-reported working conditions in 2010 and epigenetic age measures in 2016. The displayed effect sizes are the coefficients from linear regressions along with the 95% confidence intervals around these coefficients. The reference occupational category is managerial/professional and the coefficients for the other categories indicate the estimated difference in the epigenetic age measure between that category and managerial/professional after accounting for the other included covariates. Model 1 adjusts for age and sex. Model 2 additionally includes race/ethnicity and educational attainment. Model 3 additionally includes smoking status, alcohol consumption, physical inactivity, and Body Mass Index (BMI) category.

In the last set of regressions, shown in Figure 4, we included both occupational categorizations and self-reported working conditions in the same models. The coefficients for both the occupational categories and working conditions are similar to those in the previous sets of models. High stress at work has stronger associations with PCGrimAge (0.74 years) and DunedinPACE (0.02-year per year faster pace of ageing), while high physical effort has weaker associations with these measures than in the prior versions of these models that did not account for occupational category. As with the previous sets of models, few occupational characteristics are associated with EAA on these clocks after adjustment for lifestyle-related risk factors for EAA: only the associations between sales/clerical work and the PCHorvath (0.79 years) and PCHannum (1.08 years) clocks and between service work and PCGrimAge (0.66 years) remain statistically significant.

### Supplementary analyses

In addition to the main specification predicting epigenetic age measures from the category of current occupation, we considered an alternative specification in which we categorized individuals according to their longest occupation reported to the HRS by the 2010 wave. Appendix B presents the results from models based on the longest occupation reported to the HRS. These results (Table B3) are largely similar to those based on current occupation category, in that service and manual work show strong associations with epigenetic ageing on the second- and third-generation clocks. As in the main analyses, these associations are typically attenuated after adjustment for sociodemographic characteristics and health behaviours.

As an alternative to the self-reported degree of stress involved at one’s job, we considered high job strain, which indicates that a worker experiences high job demands but low job control and is thought to better identify workers at risk of adverse psychological consequences than the demands of the job alone [33] (Appendix C). Although this measure is commonly used in the psychology literature and has been associated with the types of health measures on which the second- and third-generation clocks are trained, we do not consider job strain as our main specification because it is constructed from questions that are asked in the HRS psychosocial leave-behind questionnaire and approximately 30% of our analytic sample either did not return this leave-behind questionnaire or did not answer enough of the questionnaire for us to determine their level of job strain. In the subset of respondents with measures of job strain, 42% were classified as having a high job strain (above median demands and below median control), approximately double the percentage strongly agreeing that their job involved lots of stress. Despite the differences between these measures, we found associations between high job strain and PCGrimAge and DunedinPACE that were of similar magnitude and significance as the associations between high job stress and these clocks (Table C2). However, the associations between high job strain and PCPhenoAge were of greater magnitude than those using job stress and were significant in both the minimally-adjusted model and model controlling for race/ethnicity and education. In these models with job strain as the measure of psychosocial stress, high physical effort was significantly associated with EAA on the PCHannum clock in all three models, in contrast to the main analyses in which no self-reported working conditions were associated with this clock.

## Discussion

This study examined associations between occupational characteristics of older adults and five measures of epigenetic ageing in a population-based sample of older adults in the United States employed in a variety of industries and occupations. We find evidence to suggest that individuals working in or those who have a history of working in sales/clerical, service, and manual work show evidence of EAA, but for the most part, these associations are substantially diminished and most are no longer significant after accounting for race/ethnicity and education.

We find that there are stronger associations in the expected directions with the second- and third-generation epigenetic clocks than with the first-generation epigenetic clocks. This is consistent with prior research finding that later-generation clocks show stronger and more consistent associations with social factors than do first-generation clocks [3,4,8]. With the first-generation PCHorvath and PCHannum clocks, sales/clerical was the only occupation category significantly associated with EAA, which was unexpected because service and manual work have been associated with worse self-rated health and increased risk of age-related disease in previous research [16–18]. In contrast, with the later-generation clocks, the minimally adjusted models reveal that sales/clerical, service, and manual occupations all exhibit accelerated ageing relative to managerial/professional occupations. With the PCGrimAge clock, service work remains a significant predictor of EAA even in the fully-adjusted model, consistent with prior work finding that service workers experience accelerated biological ageing as measured through a set of physiologic biomarkers [24].

Previous findings regarding occupational patterns of epigenetic ageing have been mixed and dependent on the epigenetic clock studied, but as with our study, when there are significant associations, they tend to be with the later-generation clocks [6,10,25,26,34,35]. Austin et al. [34] found no difference in epigenetic ageing on the PCHorvath clock between adults employed in service/manual work compared to managerial/professional work. Freni-Sterrantino et al. [25] found that white collar Finnish workers displayed signs of decelerated epigenetic ageing compared to blue collar workers with GrimAge and DunedinPoAm, but not with the Horvath, Hannum or PhenoAge measures. Using the UK Understanding Society study, Freni-Sterrantino et al. [26] observed decelerated epigenetic ageing among workers in professional and managerial jobs compared to those in routine occupations with the PhenoAge, GrimAge, DunedinPoAm, and Dunedin PACE measures but not the first-generation clocks. George et al. [6] studied British workers at age 53 and found no association between respondent’s occupation and the Horvath or Hannum clocks, an association between unskilled manual labour and accelerated PhenoAge, and a clear occupational gradient in accelerated GrimAge, with more disadvantaged occupations exhibiting greater degrees of age acceleration. McCrory et al. [35] found no association between current occupational position and the Horvath, Hannum, or PhenoAge clocks among Irish adults over age 50 in The Irish Longitudinal Study on Ageing (TILDA). Schmitz et al. [10] studied the association between occupational prestige and epigenetic ageing among participants in the HRS and the Multi-Ethnic Study of Atherosclerosis (MESA), using General Social Survey rankings of occupational prestige, finding that lower occupational prestige was significantly associated with GrimAge and DunedinPoAm EAA but not PhenoAge.

With few exceptions, we did not find the self-reported working conditions of high physical effort, high job stress, and long working hours to be as consistently associated with epigenetic ageing. We did find high physical effort and high stress to be associated with accelerated PCGrimAge and DunedinPACE, which is consistent with prior work demonstrating that these working conditions are risk factors for the types of biomarkers and health outcomes on which these epigenetic measures were trained, although this would also be true for PCPhenoAge [24,36,37]. However, these associations were more modest than those of the occupational categories, which could reflect the fact that occupational categories capture numerous working conditions, exposures, and benefits beyond the specific conditions examined in this study. These associations generally did not remain significant after adjustments for social factors and health behaviours, suggesting that occupational physical activity may appear to be a risk factor for EAA through its associations with socioeconomic status while stress at work may be a risk factor for EAA through its associations with health behaviours outside of work. A prior study based on the FinnTwin12 cohort of Finnish twins similarly found that occupational physical activity among older adults (aged 55–74) was associated with accelerated epigenetic ageing on the GrimAge clock when adjusting for age and sex, but that this association no longer persisted after adjustments for BMI [27]. However, this same study found stronger, more persistent associations among a younger cohort of adults in their 20s, suggesting that working conditions may influence epigenetic ageing earlier in the life course. Another prior study based on a Finnish birth cohort examined associations between several working conditions at age 46 and the same five clocks as in the present study [25]. Their study also found that occupational physical activity was associated with EEA on the GrimAge clock that did not persist after similar adjustments; however, this study found long working hours to be associated with EAA on the Horvath and Hannum clocks even after adjustment for a similar set of covariates as used in the present study [25]. Freni-Sterrantino et al. [26], examined work hours and occupational physical activity in relation to these same clocks, finding that full-time (40 hours per week) workers showed evidence of EAA on PhenoAge relative to part-time workers (less than 40 hours per week) and that high occupational physical activity was associated with EAA on GrimAge and DunedinPACE, but only the association between occupational physical activity and DunedinPACE remained after adjustment for a similar set of covariates as in our study.

Last, we jointly considered the occupational categories and the self-reported working conditions as predictors of the epigenetic age measures. Although high work stress was a significant predictor of PCGrimAge and DunedinPACE, working conditions do not appear to explain why individuals employed in lower status occupations display EEA, because in the minimally adjusted models, the occupational categorizations remain strongly associated with the second- and third-generation clocks even in the presence of these self-reported working conditions. As with the previous analyses, the initial associations are largely attenuated after adjustment for social characteristics, and only a few associations remain significant after adjustment for health behaviours.

This study contains several limitations. Because we have epigenetic measures available at only one time point for respondents when they are age 56 and above, it is not clear when epigenetic modifications occurred among our sample respondents relative to the occupational characteristics we study. Our measures of occupational characteristics are taken from jobs held around midlife, but prior work has emphasized the importance of early life disadvantage for epigenetic ageing [3,34,38–40]. To address this issue, we have considered the longest-reported occupation among jobs reported to the HRS, but we acknowledge that this does not necessarily capture the longest-held occupation over respondent’s careers. In addition to data limitations regarding the timing of measurement of epigenetic age and occupational characteristics, we are also limited by the occupational characteristics available with the HRS data, which do not include important risk factors for epigenetic ageing identified in prior literature. For example, night shift work has been associated with accelerated epigenetic ageing and methylation at specific CpG sites related to circadian rhythm [25,26,41–43]. Future work should assess these associations at younger ages, take advantage of longitudinal epigenetic age measurements, and examine additional occupational characteristics to overcome these limitations.

Despite these limitations, our study adds to the literature on work as a social determinant of accelerated ageing. Our findings suggest that occupational characteristics at midlife are associated with accelerated epigenetic ageing on second- and third-generation epigenetic clocks, but that these associations are largely accounted for by the relationships between occupational characteristics and sociodemographic characteristics of individuals. More work is needed to understand when in the life course and the specific mechanisms through which these associations occur.

## Supplementary Material

Supplemental Material

## Data Availability

The data that support the findings of this study are available from the Health and Retirement Study (HRS). These data are available to registered users who meet security requirements and agree to data use conditions specified by the HRS at https://hrsdata.isr.umich.edu/data-products/conditions-of-use

## Note

1. The code to estimate the PC clocks is available from the Levine Lab github: https://github.com/MorganLevineLab/PC-Clocks. Because DunedinPACE already excludes CpG sites with low reliability, there is not an additional PC version of this measure.

## Appendix A. Regression tables for main analyses

**Table A1.** Associations between occupational category and epigenetic age measures.

	PCHorvath			PCHannum			PCPhenoAge			PCGrimAge			DunedinPACE		
	M1	M2	M3	M1	M2	M3	M1	M2	M3	M1	M2	M3	M1	M2	M3
Managerial/															
Professional (reference)															
Sales/Clerical	1.025**	0.835*	0.831*	1.327***	1.120**	1.141**	1.551***	0.681	0.716**^+^**	0.856***	0.233	0.256	0.033**	0.006	0.006
p-value	0.005	0.029	0.028	<0.001	0.007	0.005	<0.001	0.112	0.08	<0.001	0.384	0.261	0.001	0.584	0.511
Service	0.44	0.717	0.518	0.883^+^	0.977^+^	0.759	2.442***	1.101*	0.761	1.546***	0.879**	0.616*	0.05***	−0.001	−0.007
p-value	0.319	0.129	0.27	0.062	0.057	0.135	<0.001	0.038	0.131	<0.001	0.008	0.028	<0.001	0.921	0.57
Manual	0.342	0.038	0.009	0.441	0.089	0.158	1.913***	0.564	0.616	1.993***	1.049**	0.384	0.052***	0.005	−0.003
p-value	0.419	0.935	0.985	0.334	0.861	0.756	<0.001	0.282	0.222	<0.001	0.001	0.171	<0.001	0.681	0.799
Controls for age & sex?	X	X	X	X	X	X	X	X	X	X	X	X	X	X	X
Controls for race & education?		X	X		X	X		X	X		X	X		X	X
Controls for health			X			X			X			X			X
behaviors in 2016?															
R-squared	0.242	0.277	0.3	0.248	0.266	0.297	0.3	0.334	0.407	0.469	0.494	0.645	0.036	0.119	0.259
AIC	8,036	7,988	7,964	8,219	8,201	8,164	8,324	8,276	8,145	7,131	7,084	6,656	−947	−1,048	−1,249
N	1,251	1,251	1,251	1,251	1,251	1,251	1,251	1,251	1,251	1,251	1,251	1,251	1,251	1,251	1,251

^+^p < 0.10, * p < 0.05, ** p < 0.01, *** p <0.001. Results are pooled across 10 imputations. Model 1 adjusts for age and sex. Model 2 additionally includes race/ethnicity and educational attainment. Model 3 additionally includes smoking status, alcohol consumption, physical inactivity, and Body Mass Index (BMI) category.

**Table A2.** Associations between self-reported working conditions and epigenetic age measures.

	PCHorvath			PCHannum			PCPhenoAge			PCGrimAge			DunedinPACE		
	M1	M2	M3	M1	M2	M3	M1	M2	M3	M1	M2	M3	M1	M2	M3
High stress	−0.175	−0.294	−0.413	−0.276	−0.337	−0.448	−0.248	0.029	−0.227	0.473^+^	0.582*	0.178	0.012	0.022*	0.008
p-value	0.623	0.401	0.236	0.472	0.377	0.238	0.542	0.941	0.545	0.061	0.017	0.388	0.226	0.018	0.339
High physical effort	0.097	0.133	0.193	0.559	0.515	0.58	0.477	−0.384	−0.319	0.564*	0.031	−0.025	0.027*	0	−0.002
p-value	0.802	0.733	0.616	0.182	0.226	0.165	0.277	0.378	0.44	0.04	0.908	0.912	0.01	0.968	0.81
Long working hours	0.26	0.109	0.092	0.255	0.151	0.112	−0.087	0.177	0.169	0.518	0.615^+^	0.26	0.001	0.009	0.001
p-value	0.592	0.82	0.847	0.628	0.774	0.83	0.873	0.739	0.741	0.131	0.063	0.363	0.969	0.496	0.916
Controls for age & sex?	X	X	X	X	X	X	X	X	X	X	X	X	X	X	X
Controls for race & education?		X	X		X	X		X	X		X	X		X	X
Controls for health			X			X			X			X			X
behaviors in 2016?															
R-squared	0.237	0.274	0.298	0.243	0.262	0.293	0.281	0.331	0.406	0.449	0.492	0.644	0.018	0.123	0.259
AIC	8,043	7,994	7,968	8,229	8,208	8,170	8,359	8,280	8,148	7,178	7,087	6,660	−925	−1,054	−1,248
N	1,251	1,251	1,251	1,251	1,251	1,251	1,251	1,251	1,251	1,251	1,251	1,251	1,251	1,251	1,251

^+^p < 0.10, * p < 0.05, ** p < 0.01, *** p <0.001. Results are pooled across 10 imputations. Model 1 adjusts for age and sex. Model 2 additionally includes race/ethnicity and educational attainment. Model 3 additionally includes smoking status, alcohol consumption, physical inactivity, and Body Mass Index (BMI) category.

**Table A3.** Joint association between current occupation category and self-reported working conditions in 2010 and epigenetic age measures.

	PCHorvath			PCHannum			PCPhenoAge			PCGrimAge			DunedinPACE		
	M1	M2	M3	M1	M2	M3	M1	M2	M3	M1	M2	M3	M1	M2	M3
Managerial/ Professional (reference)															
Sales/ Clerical	1.036**	0.813*	0.789*	1.298**	1.078*	1.079**	1.57***	0.731^+^	0.736^+^	0.948***	0.334	0.300	0.033**	0.008	0.007
p-value	0.005	0.034	0.039	0.001	0.010	0.009	<0.001	0.090	0.074	<0.001	0.214	0.192	0.001	0.424	0.446
Service	0.439	0.677	0.449	0.781	0.867^+^	0.62	2.489***	1.209*	0.831	1.626***	0.968**	0.659*	0.049***	0.001	−0.006
p-value	0.332	0.158	0.346	0.109	0.096	0.230	<0.001	0.025	0.105	<0.001	0.004	0.020	<0.001	0.951	0.636
Manual	0.335	−0.013	−0.079	0.33	−0.034	0.000	1.963***	0.678	0.678	2.087***	1.166***	0.440	0.052***	0.008	−0.002
p-value	0.443	0.978	0.868	0.484	0.947	1.000	<0.001	0.204	0.188	<0.001	<0.001	0.124	<0.001	0.522	0.899
High stress	−0.089	−0.249	−0.374	−0.166	−0.279	−0.386	0.075	0.127	−0.130	0.744**	0.691**	0.235	0.02*	0.023*	0.009
p-value	0.804	0.479	0.288	0.669	0.467	0.313	0.852	0.749	0.730	0.003	0.005	0.258	0.049	0.015	0.336
High physical effort	−0.013	0.094	0.18	0.403	0.467	0.55	−0.177	−0.548	−0.448	0.009	−0.171	−0.126	0.013	0.000	−0.001
p-value	0.975	0.813	0.647	0.352	0.280	0.196	0.693	0.216	0.287	0.974	0.535	0.585	0.253	0.981	0.891
Long working hours	0.335	0.161	0.152	0.35	0.222	0.189	−0.039	0.181	0.185	0.516	0.572+	0.251	0.001	0.009	0.002
p-value	0.490	0.736	0.751	0.506	0.673	0.716	0.943	0.734	0.717	0.125	0.084	0.379	0.923	0.480	0.858
Controls for age & sex?	X	X	X	X	X	X	X	X	X	X	X	X	X	X	X
Race & education?		X	X		X	X		X	X		X	X		X	X
Health behaviours in 2016?			X			X			X			X			X
R-squared	0.242	0.277	0.301	0.249	0.267	0.298	0.301	0.335	0.408	0.475	0.499	0.646	0.040	0.124	0.260
AIC	8,042	7,994	7,969	8,224	8,205	8,168	8,330	8,280	8,149	7,125	7,078	6,660	−947	−1,049	−1,244
N	1,251	1,251	1,251	1,251	1,251	1,251	1,251	1,251	1,251	1,251	1,251	1,251	1,251	1,251	1,251

^+^p < 0.10, * p < 0.05, ** p < 0.01, *** p <0.001. Results are pooled across 10 imputations. Model 1 adjusts for age and sex. Model 2 additionally includes race/ethnicity and educational attainment. Model 3 additionally includes smoking status, alcohol consumption, physical inactivity, and Body Mass Index (BMI) category.

## Appendix B. Analyses of longest occupation category reported to the HRS

In addition to the main specification predicting epigenetic age measures from the category of current occupation, we considered an alternative specification in which we categorized individuals according to the longest occupation reported to the HRS by the 2010 wave. The longest reported job tenure is constructed by RAND as the maximum tenure of a) current jobs reported in the core waves, b) the last job reported by individuals not working at their first interview, and c) the most recent job held before the current or last job that was held for 5 or more years. Because of these restrictions, the occupational category of the longest-reported job is not necessarily the occupational category of the longest-held job over the respondent’s lifetime.

**Table B1.** Summary statistics of longest-reported occupational category in analytic sample.

Number of respondents	1,246
Longest-reported occupational category in 2010^a^	
Professional/managerial	44.0%
Sales/clerical	24.6%
Service	11.6%
Manual	19.8%
Military	<1%

Note: Data are from the Health and Retirement Study. The sample is restricted to participants aged 51–64 in 2010 who reported working for pay in the 2010 HRS core wave and who subsequently participated in the 2016 Venous Blood Study (VBS) and were part of the DNA methylation assay subsample. Individual earnings are constructed by RAND and already contain imputations for missing values. Summary statistics are weighted with VBS DNA methylation sample weights. 5.4% of respondents were missing longest-reported occupation category. Missing values were imputed using multiple imputation before running the regression analyses. Respondents reporting a military occupation as their longest-held job were excluded because military occupations do not neatly fall into these categories and there are too few respondents with a history of military work for it to be a distinct category.

Table B2 below displays the distribution of current occupation category in the 2010 wave by the longest-reported occupation category in the 2010 wave. Within each category of longest-reported occupation category, the majority of individuals (upwards of 75%) are still employed in the same category. Continued employment in the same category as one’s longest-reported job is most common among workers with a history of managerial/professional employment, and least common among workers with a history of manual employment.

**Table B2.** Current occupational category by longest-reported occupational category.

	Longest-reported occupation category in 2010 wave			
	Managerial/professional	Sales/clerical	Service	Manual
Current occupation category in 2010 wave				
Managerial/professional	9.1	10.2	4.2	5.5
Sales/clerical	5.6	81.0	10.3	6.9
Service	1.8	6.6	77.7	11.7
Manual	2.4	2.2	7.8	76.0
Total	100%	100%	100%	100%

**Table B3.** Associations between longest-reported occupational category and epigenetic age measures.

	PCHorvath			PCHannum			PCPhenoAge			PCGrimAge			DunedinPACE		
	M1	M2	M3	M1	M2	M3	M1	M2	M3	M1	M2	M3	M1	M2	M3
Managerial/															
Professional (reference)															
Sales/Clerical	0.612	0.508	0.421	1.121**	0.993*	0.934*	1.784***	1.026*	0.957*	0.669*	0.171	0.097	0.031**	0.007	0.007
Service	0.339	0.598	0.322	1.084*	1.171*	0.916^+^	2.792***	1.504*	1.052^+^	1.765***	1.138**	0.57^+^	0.058***	0.008	−0.003
Manual	0.204	−0.143	−0.348	0.272	−0.121	−0.304	2.24***	0.956^+^	0.584	1.968***	1.068**	0.33	0.066***	0.019	0.002
Controls for age & sex?	X	X	X	X	X	X	X	X	X	X	X	X	X	X	X
Controls for race & education?		X	X		X	X		X	X		X	X		X	X
Controls for health			X			X			X			X			X
behaviors in 2016?															
N	1,246	1,246	1,246	1,246	1,246	1,246	1,246	1,246	1,246	1,246	1,246	1,246	1,246	1,246	1,246

^+^p < 0.10, * p < 0.05, ** p < 0.01, *** p <0.001. Results are pooled across 10 imputations. Model 1 adjusts for age and sex. Model 2 additionally includes race/ethnicity and educational attainment. Model 3 additionally includes smoking status, alcohol consumption, physical inactivity, and Body Mass Index (BMI) category.

## Appendix C. Analyses of job strain as measure of job stress

As an alternative measure of job stress, we considered job strain, which is a concept in the psychology literature defined by the combination of job demands and job control. Unlike the question about overall job stress that is asked of every working respondent in the core HRS waves, these questions related to job demands and job control are asked as part of the leave-behind questionnaire, which respondents must fill out and mail back on their own. The leave-behind questionnaire is administered to alternating halves of the sample in each wave, so for individuals in our analytic sample who were not assigned the leave-behind questionnaire in 2010, we took their responses from 2008 or 2012. Approximately 30% of the sample did not respond to the leave-behind questionnaire in any of these waves or did not respond to enough of the questions necessary to construct our measure of job strain.

For the subset of our analytic sample who responded to the leave-behind questionnaire in one of these waves, we constructed summary indices of job demands and job control based on the average of the variables described in Table C1. Where necessary, we reverse coded variables within the indices so that higher scores indicate greater job demands or greater job control. In cases where respondents were missing individual items within job demands or within job control, we calculated the average of the items for which they had non-missing responses.

**Table C1.** Summary of job demands and job control questionnaire items.

Variable	Question wording	Possible responses	Coding
Job demands items			
Time pressure	I am under constant time pressure due to a heavy workload.	1 = strongly disagree 2 = disagree 3 = agree 4 = strongly agree	Kept original coding scale
Working fast	Considering the things I have to do at work, I have to work very fast.	1 = strongly disagree 2 = disagree 3 = agree 4 = strongly agree	Kept original coding scale
Conflicting demands	In my work I am free from conflicting demands that others make.	1 = strongly disagree 2 = disagree 3 = agree 4 = strongly agree	Reverse coded such that 1 = strongly agree and 4 = strongly disagree.
Job control items			
Freedom over work	I have very little freedom to decide how I do my work.	1 = strongly disagree 2 = disagree 3 = agree 4 = strongly agree	Reverse coded such that 1 = strongly agree and 4 = strongly disagree.
Control over what happens	At work, I feel I have control over what happens in most situations.	1 = strongly disagree 2 = disagree 3 = agree 4 = strongly agree	Kept original coding scale
Opportunity to develop skills	I have the opportunity to develop new skills.	1 = strongly disagree 2 = disagree 3 = agree 4 = strongly agree	Kept original coding scale

After creating the indices of job demands and job control, we dichotomized both at their sample medians and individuals with above-median job demands and below-median job control were classified as having high job strain [44]. 42% of the respondents who participated in the leave-behind questionnaire were classified as experiencing high job strain.

The results of models predicting the epigenetic age measures from this measure of high job strain, high physical effort, and long working hours are displayed in Table C2.

**Table C2.** Associations between self-reported working conditions and epigenetic age measures in subsample that responded to psychosocial leave-behind questionnaire: High job strain as measure of job stress.

	PCHorvath			PCHannum			PCPhenoAge			PCGrimAge			DunedinPACE		
	M1	M2	M3	M1	M2	M3	M1	M2	M3	M1	M2	M3	M1	M2	M3
High job strain	0.427	0.334	0.268	0.54	0.468	0.42	0.834*	0.864*	0.715^+^	0.469*	0.461*	0.251	0.023*	0.025**	0.018*
High physical effort	0.603	0.607	0.669	1.256*	1.209*	1.315*	0.766	−0.092	−0.032	0.757*	0.334	0.243	0.018	−0.009	−0.011
Long work hours	0.237	0.084	−0.015	−0.011	−0.112	−0.176	−0.822	−0.392	−0.353	0.216	0.37	−0.059	−0.008	0.006	−0.002
Controls for age & sex?	X	X	X	X	X	X	X	X	X	X	X	X	X	X	X
Controls for race & education?		X	X		X	X		X	X		X	X		X	X
Controls for health			X			X			X			X			X
behaviors in 2016?															
N	886	886	886	886	886	886	886	886	886	886	886	886	886	886	886

^+^p < 0.10, * p < 0.05, ** p < 0.01, *** p <0.001. Results are pooled across 10 imputations. Model 1 adjusts for age and sex. Model 2 additionally includes race/ethnicity and educational attainment. Model 3 additionally includes smoking status, alcohol consumption, physical inactivity, and Body Mass Index (BMI) category.

**Appendix References**

Fransson, E.I., Nyberg, S.T., Heikkilä, K., Alfredsson, L., Bacquer, D.D., Batty, G.D., Bonenfant, S., Casini, A., Clays, E., Goldberg, M., Kittel, F., Koskenvuo, M., Knutsson, A., Leineweber, C., Magnusson Hanson, L.L., Nordin, M., Singh-Manoux, A., Suominen, S., Vahtera, J., Westerholm, P., Westerlund, H., Zins, M., Theorell, T., Kivimäki, M., 2012. Comparison of alternative versions of the job demand-control scales in 17 European cohort studies: the IPD-Work consortium. BMC Public Health 12, 62. https://doi.org/10.1186/1471-2458-12-62

Karasek, R.A., 1979. Job Demands, Job Decision Latitude, and Mental Strain: Implications for Job Redesign. Administrative Science Quarterly 24, 285–308. https://doi.org/10.2307/2392498
