# Supplementary material for: Occupational characteristics and epigenetic aging among older adults in the United States
Source: Epigenetics. 2023 Jun 10;18(1):2218763. doi: 10.1080/15592294.2023.2218763 (PMC10259313; doi:10.1080/15592294.2023.2218763)
Supplement: Supplemental Material [file KEPI_A_2218763_SM1111.docx]

**Appendix**

| A1. Regression tables for main analyses | 2 |
| --- | --- |
| A2. Analyses of longest occupation category reported to the HRS | 5 |
| A3. Analyses of job strain as measure of job stress | 7 |
| References | 10 |

**Table A1.1: Associations between occupational category and epigenetic age measures**

|  | **PCHorvath** | | | **PCHannum** | | | **PCPhenoAge** | | | **PCGrimAge** | | | **DunedinPACE** | | |
| --- | --- | --- | --- | --- | --- | --- | --- | --- | --- | --- | --- | --- | --- | --- | --- |
|  | **M1** | **M2** | **M3** | **M1** | **M2** | **M3** | **M1** | **M2** | **M3** | **M1** | **M2** | **M3** | **M1** | **M2** | **M3** |
| Managerial/  Professional (reference) |  |  |  |  |  |  |  |  |  |  |  |  |  |  |  |
| Sales/Clerical | 1.025^**^ | 0.835^*^ | 0.831^*^ | 1.327^***^ | 1.120^**^ | 1.141^**^ | 1.551^***^ | 0.681 | 0.716^+^ | 0.856^***^ | 0.233 | 0.256 | 0.033^**^ | 0.006 | 0.006 |
| p-value | 0.005 | 0.029 | 0.028 | <0.001 | 0.007 | 0.005 | <0.001 | 0.112 | 0.080 | <0.001 | 0.384 | 0.261 | 0.001 | 0.584 | 0.511 |
| Service | 0.440 | 0.717 | 0.518 | 0.883^+^ | 0.977^+^ | 0.759 | 2.442^***^ | 1.101^*^ | 0.761 | 1.546^***^ | 0.879^**^ | 0.616^*^ | 0.05^***^ | -0.001 | -0.007 |
| p-value | 0.319 | 0.129 | 0.270 | 0.062 | 0.057 | 0.135 | <0.001 | 0.038 | 0.131 | <0.001 | 0.008 | 0.028 | <0.001 | 0.921 | 0.570 |
| Manual | 0.342 | 0.038 | 0.009 | 0.441 | 0.089 | 0.158 | 1.913^***^ | 0.564 | 0.616 | 1.993^***^ | 1.049^**^ | 0.384 | 0.052^***^ | 0.005 | -0.003 |
| p-value | 0.419 | 0.935 | 0.985 | 0.334 | 0.861 | 0.756 | <0.001 | 0.282 | 0.222 | <0.001 | 0.001 | 0.171 | <0.001 | 0.681 | 0.799 |
| Controls for age & sex? | X | X | X | X | X | X | X | X | X | X | X | X | X | X | X |
| Controls for race & education? |  | X | X |  | X | X |  | X | X |  | X | X |  | X | X |
| Controls for health  behaviors in 2016? |  |  | X |  |  | X |  |  | X |  |  | X |  |  | X |
| R-squared | 0.242 | 0.277 | 0.300 | 0.248 | 0.266 | 0.297 | 0.300 | 0.334 | 0.407 | 0.469 | 0.494 | 0.645 | 0.036 | 0.119 | 0.259 |
| AIC | 8,036 | 7,988 | 7,964 | 8,219 | 8,201 | 8,164 | 8,324 | 8,276 | 8,145 | 7,131 | 7,084 | 6,656 | -947 | -1,048 | -1,249 |
| N | 1,251 | 1,251 | 1,251 | 1,251 | 1,251 | 1,251 | 1,251 | 1,251 | 1,251 | 1,251 | 1,251 | 1,251 | 1,251 | 1,251 | 1,251 |

^+^ p < 0.10, * p < 0.05, ** p < 0.01, *** p <0.001

Results are pooled across 10 imputations. Model 1 adjusts for age and sex. Model 2 additionally includes race/ethnicity and educational attainment. Model 3 additionally includes smoking status, alcohol consumption, physical inactivity, and Body Mass Index (BMI) category.

**Table A1.2: Associations between self-reported working conditions and epigenetic age measures**

|  | **PCHorvath** | | | **PCHannum** | | | **PCPhenoAge** | | | **PCGrimAge** | | | **DunedinPACE** | | |
| --- | --- | --- | --- | --- | --- | --- | --- | --- | --- | --- | --- | --- | --- | --- | --- |
|  | **M1** | **M2** | **M3** | **M1** | **M2** | **M3** | **M1** | **M2** | **M3** | **M1** | **M2** | **M3** | **M1** | **M2** | **M3** |
| High stress | -0.175 | -0.294 | -0.413 | -0.276 | -0.337 | -0.448 | -0.248 | 0.029 | -0.227 | 0.473^+^ | 0.582^*^ | 0.178 | 0.012 | 0.022^*^ | 0.008 |
| p-value | 0.623 | 0.401 | 0.236 | 0.472 | 0.377 | 0.238 | 0.542 | 0.941 | 0.545 | 0.061 | 0.017 | 0.388 | 0.226 | 0.018 | 0.339 |
| High physical effort | 0.097 | 0.133 | 0.193 | 0.559 | 0.515 | 0.580 | 0.477 | -0.384 | -0.319 | 0.564^*^ | 0.031 | -0.025 | 0.027^*^ | 0.000 | -0.002 |
| p-value | 0.802 | 0.733 | 0.616 | 0.182 | 0.226 | 0.165 | 0.277 | 0.378 | 0.440 | 0.040 | 0.908 | 0.912 | 0.010 | 0.968 | 0.810 |
| Long working hours | 0.260 | 0.109 | 0.092 | 0.255 | 0.151 | 0.112 | -0.087 | 0.177 | 0.169 | 0.518 | 0.615^+^ | 0.260 | 0.001 | 0.009 | 0.001 |
| p-value | 0.592 | 0.820 | 0.847 | 0.628 | 0.774 | 0.830 | 0.873 | 0.739 | 0.741 | 0.131 | 0.063 | 0.363 | 0.969 | 0.496 | 0.916 |
| Controls for age & sex? | X | X | X | X | X | X | X | X | X | X | X | X | X | X | X |
| Controls for race & education? |  | X | X |  | X | X |  | X | X |  | X | X |  | X | X |
| Controls for health  behaviors in 2016? |  |  | X |  |  | X |  |  | X |  |  | X |  |  | X |
| R-squared | 0.237 | 0.274 | 0.298 | 0.243 | 0.262 | 0.293 | 0.281 | 0.331 | 0.406 | 0.449 | 0.492 | 0.644 | 0.018 | 0.123 | 0.259 |
| AIC | 8,043 | 7,994 | 7,968 | 8,229 | 8,208 | 8,170 | 8,359 | 8,280 | 8,148 | 7,178 | 7,087 | 6,660 | -925 | -1,054 | -1,248 |
| N | 1,251 | 1,251 | 1,251 | 1,251 | 1,251 | 1,251 | 1,251 | 1,251 | 1,251 | 1,251 | 1,251 | 1,251 | 1,251 | 1,251 | 1,251 |

^+^ p < 0.10, * p < 0.05, ** p < 0.01, *** p <0.001

Results are pooled across 10 imputations. Model 1 adjusts for age and sex. Model 2 additionally includes race/ethnicity and educational attainment. Model 3 additionally includes smoking status, alcohol consumption, physical inactivity, and Body Mass Index (BMI) category.

**Table A1.3: Joint association between current occupation category and self-reported working conditions in 2010 and epigenetic age measures**

|  | **PCHorvath** | | | **PCHannum** | | | **PCPhenoAge** | | | **PCGrimAge** | | | **DunedinPACE** | | |
| --- | --- | --- | --- | --- | --- | --- | --- | --- | --- | --- | --- | --- | --- | --- | --- |
|  | **M1** | **M2** | **M3** | **M1** | **M2** | **M3** | **M1** | **M2** | **M3** | **M1** | **M2** | **M3** | **M1** | **M2** | **M3** |
| Managerial/  Professional (reference) |  |  |  |  |  |  |  |  |  |  |  |  |  |  |  |
| Sales/  Clerical | 1.036^**^ | 0.813^*^ | 0.789^*^ | 1.298^**^ | 1.078^*^ | 1.079^**^ | 1.57^***^ | 0.731^+^ | 0.736^+^ | 0.948^***^ | 0.334 | 0.300 | 0.033^**^ | 0.008 | 0.007 |
| p-value | 0.005 | 0.034 | 0.039 | 0.001 | 0.010 | 0.009 | <0.001 | 0.090 | 0.074 | <0.001 | 0.214 | 0.192 | 0.001 | 0.424 | 0.446 |
| Service | 0.439 | 0.677 | 0.449 | 0.781 | 0.867^+^ | 0.62 | 2.489^***^ | 1.209^*^ | 0.831 | 1.626^***^ | 0.968^**^ | 0.659^*^ | 0.049^***^ | 0.001 | -0.006 |
| p-value | 0.332 | 0.158 | 0.346 | 0.109 | 0.096 | 0.230 | <0.001 | 0.025 | 0.105 | <0.001 | 0.004 | 0.020 | <0.001 | 0.951 | 0.636 |
| Manual | 0.335 | -0.013 | -0.079 | 0.33 | -0.034 | 0.000 | 1.963^***^ | 0.678 | 0.678 | 2.087^***^ | 1.166^***^ | 0.440 | 0.052^***^ | 0.008 | -0.002 |
| p-value | 0.443 | 0.978 | 0.868 | 0.484 | 0.947 | 1.000 | <0.001 | 0.204 | 0.188 | <0.001 | <0.001 | 0.124 | <0.001 | 0.522 | 0.899 |
| High stress | -0.089 | -0.249 | -0.374 | -0.166 | -0.279 | -0.386 | 0.075 | 0.127 | -0.130 | 0.744^**^ | 0.691^**^ | 0.235 | 0.02^*^ | 0.023^*^ | 0.009 |
| p-value | 0.804 | 0.479 | 0.288 | 0.669 | 0.467 | 0.313 | 0.852 | 0.749 | 0.730 | 0.003 | 0.005 | 0.258 | 0.049 | 0.015 | 0.336 |
| High physical effort | -0.013 | 0.094 | 0.18 | 0.403 | 0.467 | 0.55 | -0.177 | -0.548 | -0.448 | 0.009 | -0.171 | -0.126 | 0.013 | 0.000 | -0.001 |
| p-value | 0.975 | 0.813 | 0.647 | 0.352 | 0.280 | 0.196 | 0.693 | 0.216 | 0.287 | 0.974 | 0.535 | 0.585 | 0.253 | 0.981 | 0.891 |
| Long working hours | 0.335 | 0.161 | 0.152 | 0.35 | 0.222 | 0.189 | -0.039 | 0.181 | 0.185 | 0.516 | 0.572+ | 0.251 | 0.001 | 0.009 | 0.002 |
| p-value | 0.490 | 0.736 | 0.751 | 0.506 | 0.673 | 0.716 | 0.943 | 0.734 | 0.717 | 0.125 | 0.084 | 0.379 | 0.923 | 0.480 | 0.858 |
| Controls for age & sex? | X | X | X | X | X | X | X | X | X | X | X | X | X | X | X |
| Race & education? |  | X | X |  | X | X |  | X | X |  | X | X |  | X | X |
| Health behaviors in 2016? |  |  | X |  |  | X |  |  | X |  |  | X |  |  | X |
| R-squared | 0.242 | 0.277 | 0.301 | 0.249 | 0.267 | 0.298 | 0.301 | 0.335 | 0.408 | 0.475 | 0.499 | 0.646 | 0.040 | 0.124 | 0.260 |
| AIC | 8,042 | 7,994 | 7,969 | 8,224 | 8,205 | 8,168 | 8,330 | 8,280 | 8,149 | 7,125 | 7,078 | 6,660 | -947 | -1,049 | -1,244 |
| N | 1,251 | 1,251 | 1,251 | 1,251 | 1,251 | 1,251 | 1,251 | 1,251 | 1,251 | 1,251 | 1,251 | 1,251 | 1,251 | 1,251 | 1,251 |

^+^ p < 0.10, * p < 0.05, ** p < 0.01, *** p <0.001

Results are pooled across 10 imputations. Model 1 adjusts for age and sex. Model 2 additionally includes race/ethnicity and educational attainment. Model 3 additionally includes smoking status, alcohol consumption, physical inactivity, and Body Mass Index (BMI) category.

**A2. Analyses of longest occupation category reported to the HRS**

In addition to the main specification predicting epigenetic age measures from the category of current occupation, we considered an alternative specification in which we categorized individuals according to the longest occupation reported to the HRS by the 2010 wave. The longest reported job tenure is constructed by RAND as the maximum tenure of a) current jobs reported in the core waves, b) the last job reported by individuals not working at their first interview, and c) the most recent job held before the current or last job that was held for 5 or more years. Because of these restrictions, the occupational category of the longest-reported job is not necessarily the occupational category of the longest-held job over the respondent’s lifetime.

**Table A2.1 Summary statistics of longest-reported occupational category in analytic sample**

| Number of respondents | 1,246 |
| --- | --- |
| Longest-reported occupational category in 2010^a^ |  |
| Professional/managerial | 44.0 % |
| Sales/clerical | 24.6 % |
| Service | 11.6 % |
| Manual | 19.8 % |
| Military | <1% |

Note: Data are from the Health and Retirement Study. The sample is restricted to participants aged 51-64 in 2010 who reported working for pay in the 2010 HRS core wave and who subsequently participated in the 2016 Venous Blood Study (VBS) and were part of the DNA methylation assay subsample. Individual earnings are constructed by RAND and already contain imputations for missing values. Summary statistics are weighted with VBS DNA methylation sample weights.

^a^ 5.4% of respondents were missing longest-reported occupation category. Missing values were imputed using multiple imputation before running the regression analyses. Respondents reporting a military occupation as their longest-held job were excluded because military occupations do not neatly fall into these categories and there are too few respondents with a history of military work for it to be a distinct category.

Table A2.2 below displays the distribution of current occupation category in the 2010 wave by the longest-reported occupation category in the 2010 wave. Within each category of longest-reported occupation category, the majority of individuals (upwards of 75%) are still employed in the same category. Continued employment in the same category as one’s longest-reported job is most common among workers with a history of managerial/professional employment, and least common among workers with a history of manual employment.

**Table A2.2: Current occupational category by longest-reported occupational category**

|  | Longest-reported occupation category in 2010 wave | | | |
| --- | --- | --- | --- | --- |
|  | Managerial/professional | Sales/clerical | Service | Manual |
| Current occupation category in 2010 wave |  |  |  |  |
| Managerial/professional | 90.1 | 10.2 | 4.2 | 5.5 |
| Sales/clerical | 5.6 | 81.0 | 10.3 | 6.9 |
| Service | 1.8 | 6.6 | 77.7 | 11.7 |
| Manual | 2.4 | 2.2 | 7.8 | 76.0 |
| Total | 100% | 100% | 100% | 100% |

**Table A2.3: Associations between longest-reported occupational category and epigenetic age measures**

|  | **PCHorvath** | | | **PCHannum** | | | **PCPhenoAge** | | | **PCGrimAge** | | | **DunedinPACE** | | |
| --- | --- | --- | --- | --- | --- | --- | --- | --- | --- | --- | --- | --- | --- | --- | --- |
|  | **M1** | **M2** | **M3** | **M1** | **M2** | **M3** | **M1** | **M2** | **M3** | **M1** | **M2** | **M3** | **M1** | **M2** | **M3** |
| Managerial/  Professional (reference) |  |  |  |  |  |  |  |  |  |  |  |  |  |  |  |
| Sales/Clerical | 0.612 | 0.508 | 0.421 | 1.121^**^ | 0.993^*^ | 0.934^*^ | 1.784^***^ | 1.026^*^ | 0.957^*^ | 0.669^*^ | 0.171 | 0.097 | 0.031^**^ | 0.007 | 0.007 |
| Service | 0.339 | 0.598 | 0.322 | 1.084^*^ | 1.171^*^ | 0.916^+^ | 2.792^***^ | 1.504^*^ | 1.052^+^ | 1.765^***^ | 1.138^**^ | 0.57^+^ | 0.058^***^ | 0.008 | -0.003 |
| Manual | 0.204 | -0.143 | -0.348 | 0.272 | -0.121 | -0.304 | 2.24^***^ | 0.956^+^ | 0.584 | 1.968^***^ | 1.068^**^ | 0.33 | 0.066^***^ | 0.019 | 0.002 |
| Controls for age & sex? | X | X | X | X | X | X | X | X | X | X | X | X | X | X | X |
| Controls for race & education? |  | X | X |  | X | X |  | X | X |  | X | X |  | X | X |
| Controls for health  behaviors in 2016? |  |  | X |  |  | X |  |  | X |  |  | X |  |  | X |
| N | 1,246 | 1,246 | 1,246 | 1,246 | 1,246 | 1,246 | 1,246 | 1,246 | 1,246 | 1,246 | 1,246 | 1,246 | 1,246 | 1,246 | 1,246 |

^+^ p < 0.10, * p < 0.05, ** p < 0.01, *** p <0.001

Results are pooled across 10 imputations. Model 1 adjusts for age and sex. Model 2 additionally includes race/ethnicity and educational attainment. Model 3 additionally includes smoking status, alcohol consumption, physical inactivity, and Body Mass Index (BMI) category.

**A3. Analyses of job strain as measure of job stress**

As an alternative measure of job stress, we considered job strain, which is a concept in the psychology literature defined by the combination of job demands and job control. Unlike the question about overall job stress that is asked of every working respondent in the core HRS waves, these questions related to job demands and job control are asked as part of the leave-behind questionnaire, which respondents must fill out and mail back on their own. The leave-behind questionnaire is administered to alternating halves of the sample in each wave, so for individuals in our analytic sample who were not assigned the leave-behind questionnaire in 2010, we took their responses from 2008 or 2012. Approximately 30% of the sample did not respond to the leave-behind questionnaire in any of these waves or did not respond to enough of the questions necessary to construct our measure of job strain.

For the subset of our analytic sample who responded to the leave-behind questionnaire in one of these waves, we constructed summary indices of job demands and job control based on the average of the variables described in Table A3.1. Where necessary, we reverse coded variables within the indices so that higher scores indicate greater job demands or greater job control. In cases where respondents were missing individual items within job demands or within job control, we calculated the average of the items for which they had non-missing responses.

**Table A3.1 Summary of job demands and job control questionnaire items**

| **Variable** | **Question wording** | **Possible responses** | **Coding** |
| --- | --- | --- | --- |
| **Job demands items** |  |  |  |
| Time pressure | I am under constant time pressure due to a heavy workload. | 1 = strongly disagree  2 = disagree  3 = agree  4 = strongly agree | Kept original coding scale |
| Working fast | Considering the things I have to do at work, I have to work very fast. | 1 = strongly disagree  2 = disagree  3 = agree  4 = strongly agree | Kept original coding scale |
| Conflicting demands | In my work I am free from conflicting demands that others make. | 1 = strongly disagree  2 = disagree  3 = agree  4 = strongly agree | Reverse coded such that 1 = strongly agree and 4 = strongly disagree. |
| **Job control items** |  |  |  |
| Freedom over work | I have very little freedom to decide how I do my work. | 1 = strongly disagree  2 = disagree  3 = agree  4 = strongly agree | Reverse coded such that 1 = strongly agree and 4 = strongly disagree. |
| Control over what happens | At work, I feel I have control over what happens in most situations. | 1 = strongly disagree  2 = disagree  3 = agree  4 = strongly agree | Kept original coding scale |
| Opportunity to develop skills | I have the opportunity to develop new skills. | 1 = strongly disagree  2 = disagree  3 = agree  4 = strongly agree | Kept original coding scale |

After creating the indices of job demands and job control, we dichotomized both at their sample medians and individuals with above-median job demands and below-median job control were classified as having high job strain (Fransson et al., 2012). 42% of the respondents who participated in the leave-behind questionnaire were classified as experiencing high job strain.

The results of models predicting the epigenetic age measures from this measure of high job strain, high physical effort, and long working hours are displayed in Table A3.2.

**Table A3.2: Associations between self-reported working conditions and epigenetic age measures in subsample that responded to psychosocial leave-behind questionnaire: High job strain as measure of job stress**

|  | **PCHorvath** | | | **PCHannum** | | | **PCPhenoAge** | | | **PCGrimAge** | | | **DunedinPACE** | | |
| --- | --- | --- | --- | --- | --- | --- | --- | --- | --- | --- | --- | --- | --- | --- | --- |
|  | **M1** | **M2** | **M3** | **M1** | **M2** | **M3** | **M1** | **M2** | **M3** | **M1** | **M2** | **M3** | **M1** | **M2** | **M3** |
| High job strain | 0.427 | 0.334 | 0.268 | 0.540 | 0.468 | 0.420 | 0.834^*^ | 0.864^*^ | 0.715^+^ | 0.469^*^ | 0.461^*^ | 0.251 | 0.023^*^ | 0.025^**^ | 0.018^*^ |
| High physical effort | 0.603 | 0.607 | 0.669 | 1.256^*^ | 1.209^*^ | 1.315^*^ | 0.766 | -0.092 | -0.032 | 0.757^*^ | 0.334 | 0.243 | 0.018 | -0.009 | -0.011 |
| Long work hours | 0.237 | 0.084 | -0.015 | -0.011 | -0.112 | -0.176 | -0.822 | -0.392 | -0.353 | 0.216 | 0.37 | -0.059 | -0.008 | 0.006 | -0.002 |
| Controls for age & sex? | X | X | X | X | X | X | X | X | X | X | X | X | X | X | X |
| Controls for race & education? |  | X | X |  | X | X |  | X | X |  | X | X |  | X | X |
| Controls for health  behaviors in 2016? |  |  | X |  |  | X |  |  | X |  |  | X |  |  | X |
| N | 886 | 886 | 886 | 886 | 886 | 886 | 886 | 886 | 886 | 886 | 886 | 886 | 886 | 886 | 886 |

^+^ p < 0.10, * p < 0.05, ** p < 0.01, *** p <0.001

Results are pooled across 10 imputations. Model 1 adjusts for age and sex. Model 2 additionally includes race/ethnicity and educational attainment. Model 3 additionally includes smoking status, alcohol consumption, physical inactivity, and Body Mass Index (BMI) category.

**Appendix References**

Fransson, E.I., Nyberg, S.T., Heikkilä, K., Alfredsson, L., Bacquer, D.D., Batty, G.D., Bonenfant, S., Casini, A., Clays, E., Goldberg, M., Kittel, F., Koskenvuo, M., Knutsson, A., Leineweber, C., Magnusson Hanson, L.L., Nordin, M., Singh-Manoux, A., Suominen, S., Vahtera, J., Westerholm, P., Westerlund, H., Zins, M., Theorell, T., Kivimäki, M., 2012. Comparison of alternative versions of the job demand-control scales in 17 European cohort studies: the IPD-Work consortium. BMC Public Health 12, 62. https://doi.org/10.1186/1471-2458-12-62

Karasek, R.A., 1979. Job Demands, Job Decision Latitude, and Mental Strain: Implications for Job Redesign. Administrative Science Quarterly 24, 285–308. https://doi.org/10.2307/2392498
